# A New Multi-Ingredient Recipe for the Treatment of Localized Advanced Periodontal Disease following the Surgical Removal of Impacted Wisdom Teeth

**DOI:** 10.1155/2016/3847615

**Published:** 2016-04-27

**Authors:** Nabil Khzam, Adam Fell, Anthony Fisher, Paul Kim, Usman A. Khan, Mahmoud M. Bakr

**Affiliations:** ^1^DB Dental, BDS, MPhil, DClinDent, MRACDS (Perio), Perth, WA 6159, Australia; ^2^DB Dental, BSc, BDent, MJDF RCS, Perth, WA 6011, Australia; ^3^PerioHEALTH and Implants Practice, BDSc, DClinDent, Brisbane, QLD 4059, Australia; ^4^Benowa Mansions Periodontal Practice, BPharm, BDSc, DClinDent, Gold Coast, QLD 4217, Australia; ^5^Dalby Dental Clinic, BDS, MDS, PhD, Western Downs, QLD 4405, Australia; ^6^General Dental Practice, School of Dentistry and Oral Health, BDS, MDS, Griffith University, Gold Coast, QLD 4222, Australia

## Abstract

Periodontal disease is a chronic inflammation of the tooth supporting structures. It leads to bone and attachment loss which is irreversible. Extraction of horizontally impacted lower third molar (L3M) teeth may result in localized periodontal pockets at the distal aspect of the adjacent lower second molars (L2M). We present a case of a 21-year-old male who suffered from a swelling and pain around his lower right second molar following surgical removal of a mesioangular impacted lower right third molar. We showed that oral hygiene measures, surgical access, mixture of autogenous and synthetic bone graft, and guided tissue regeneration (GTR) were enough to control the problem.

## 1. Introduction

The presence of horizontally impacted L3M often leads to localized periodontal disease next to the extracted tooth which often needs surgical procedure to resolve [1, 2]. Conservative periodontal treatment in terms of scaling and root planning on the distal root surface of the tooth adjacent to the impacted wisdom tooth after the extraction may result in small benefit [3]. Some studies showed that various flap designs for the surgical removal of wisdom teeth can lead to positive outcomes in terms of periodontal pocket reduction on the distal surface of the adjacent L2M [4]. On the other hand, GTR technique utilizing bone graft and barrier membranes were shown to be the treatment of choice in the prevention of these periodontal defects [5, 6]. Bone grafts to fill the periodontal defect on the distal root surface of L2M adjacent to the extracted horizontally impacted L3M have long been discussed and revealed some good results [7, 8].

In this study, a combination of autogenous bone chips harvested from the external oblique ridge and bovine porous bone mineral Bio-Oss (Geistlich Biomaterials) were used to fill the advanced periodontal pocket on the distal side of the L2M. This mixture was used to support and maintain the space that is essential for bone regeneration as well as in the support of the overlying double bioabsorbable membrane. A collagen membrane (Bio-Gide, Geistlich Biomaterials) was used to cover the bony mixture.

The innovative technique used in this case report aimed to evaluate the efficacy of using autogenous bone chips mixed with bovine bone particles plus double layer of absorbable membrane for the treatment of advanced periodontal defect on the distal root surface of L2M adjacent to the extracted mesioangular impacted L3M.

## 2. Case Presentation

**Figure 1:**
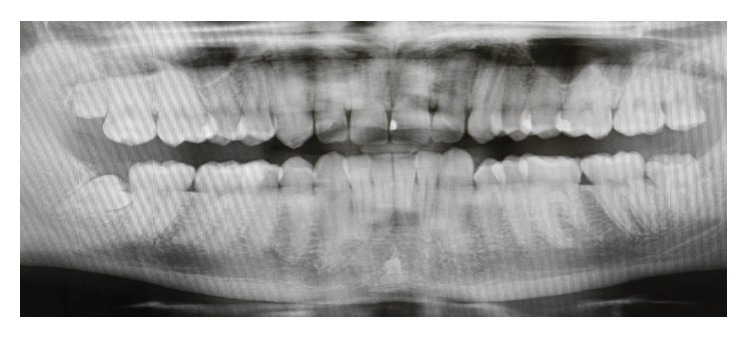
Preoperative orthopantograph (OPG) demonstrating the mesioangular impaction of the lower right third molar.

**Figure 2:**
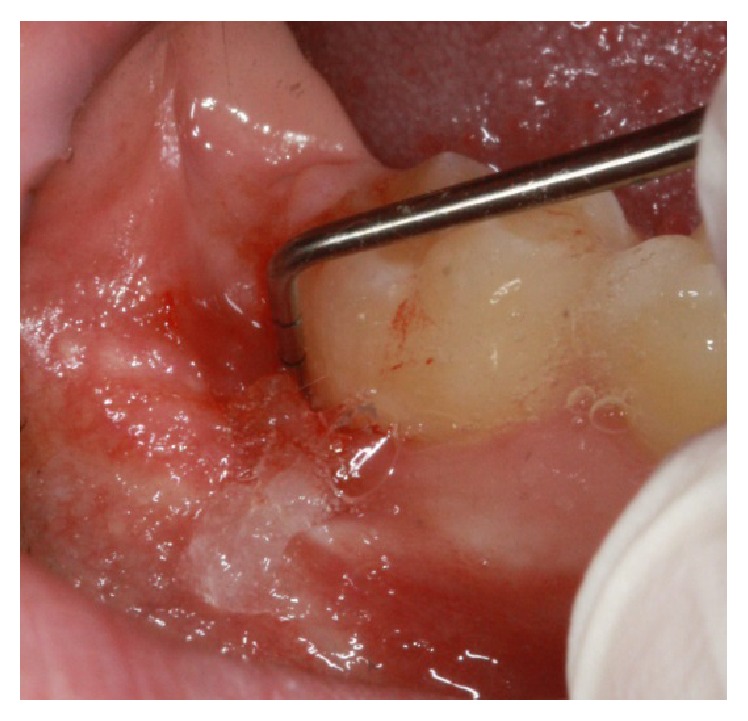
Preoperative clinical photograph of the lower right second molar.

**Figure 3:**
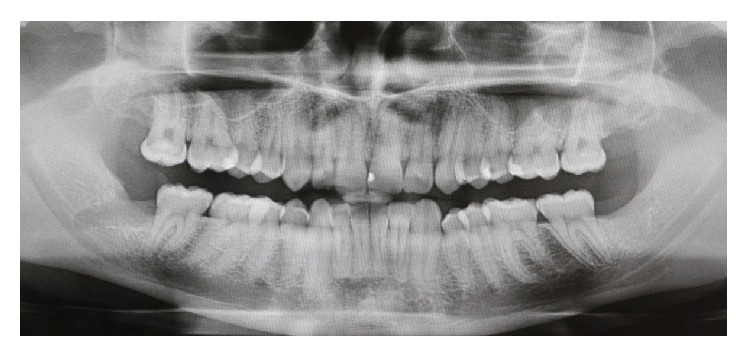
Postoperative orthopantograph (OPG) showing the extraction socket of the lower right third molar.

**Figure 4:**
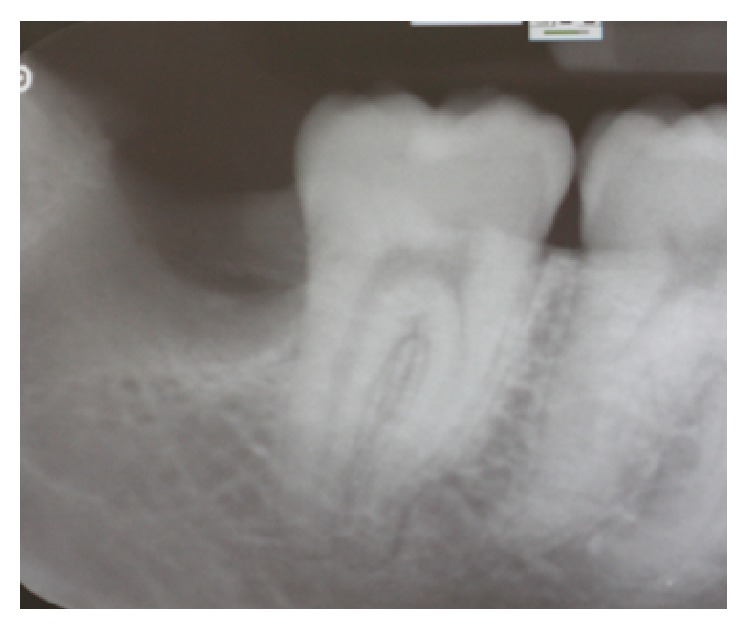
Preoperative periapical X-ray of the lower right second molar.

A healthy 21-year-old male, nonsmoker, was referred to a specialist periodontist (NK) for a consultation regarding a swelling and associated halitosis, which started three months after surgical extraction of right L3M tooth that was impacted in a mesioangular position as shown in the orthopantograph (OPG) (Figure 1). Clinical examination revealed an eight-millimeter-deep periodontal pocket, bleeding on probing, and suppuration on the distal root surface of the right L2M adjacent to the mesioangular impacted right L3M that was extracted three months ago (Figure 2). A new OPG and a periapical radiograph were taken to explore the distal root of L2M before surgery planning. Both radiographs showed incomplete healing and poor bone fill in the extraction socket of the right L3M, as well as vertical bone loss distal to the L2M (Figures 3 and 4).

**Figure 5:**
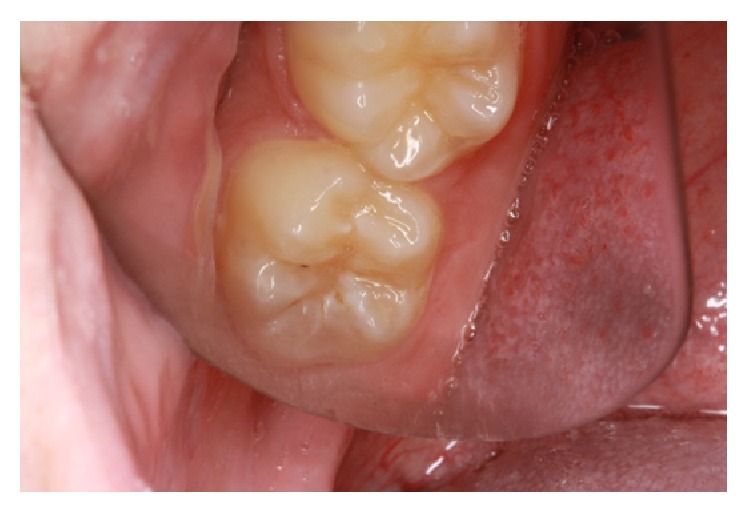
Postoperative clinical photograph of the lower right second molar.

**Figure 6:**
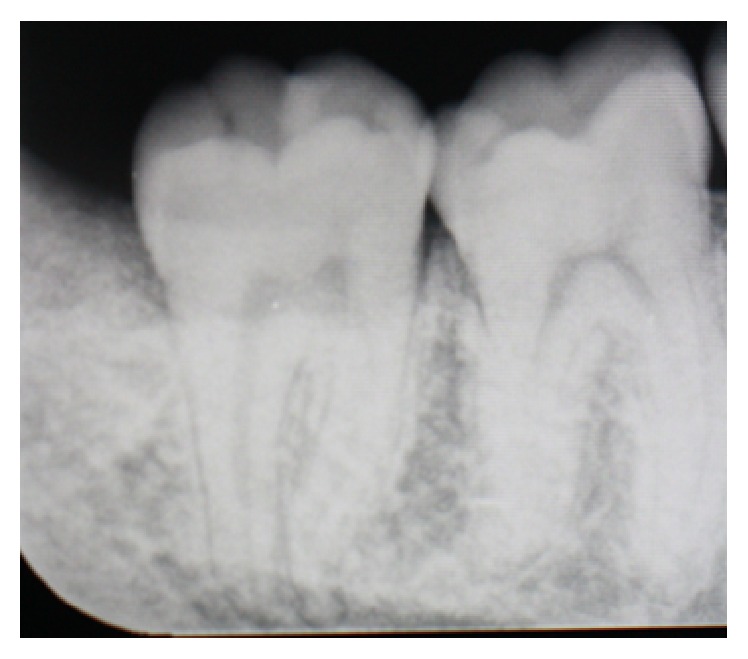
Postoperative periapical X-ray of the lower right second molar.

**Figure 7:**
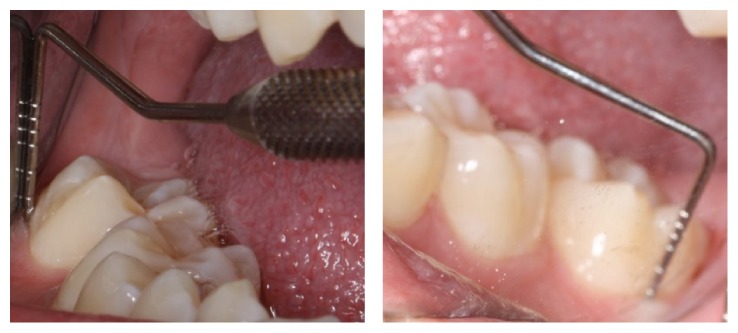
Postoperative clinical photograph of the lower right second molar confirming the pocket depth after the successful treatment.

GTR technique was conducted under local anesthesia. A full thickness mucoperiosteal flap was raised around the distal side of L2M. Chronically inflamed tissue was removed using piezo tips on a piezo surgery unit. Debridement of the distal root surface of L2M was performed using a combination of ultrasonic tips and 13/14 Gracey Curettes. A bone scraper was used to harvest bone from the external oblique ridge. A mixture of autogenous bone chips and Bio-Oss particles was used to fill the intrabony defects distal to L2M. Double layer of Bio-Gide membrane was used to cover the bone mixture, Vicrly sutures 5-0 (Ethicon GmbH, Germany) were used to secure the membrane. Prolene 5-0 sutures (Ethicon GmbH, Germany) were used to achieve primary closure. Postoperative and oral hygiene instructions were given. A period of five months was allowed for healing before reviewing the case. Upon review, the pocket depth was reduced to 3 mm with complete absence of bleeding upon probing and suppuration on the distal root surface of L2M. Patient was informed to attend regular review appointments for monitoring. Both clinical photograph and periapical X-ray were taken to confirm the good result after 4 months from the day of surgery (Figures 5, 6, and 7).

## 3. Discussion

Extraction of impacted L3M often results in increased risk for caries and periodontal intrabony defects adjacent to the distal surface of the L2M [1, 9]. A retrospective study revealed that 43% of the cases had periodontal pocket of ≥7 mm on the distal side of the adjacent L2M two years after the surgical removal of the impacted wisdom molar [2, 10]. In this retrospective report of 215 cases, the investigators were able to show that the initial periodontal defect, the angle of the impacted molar, and the age of the patient were important factors that influenced the incidence of localized advanced periodontal disease. In our case report, the advanced periodontal disease developed within 4 weeks after tooth extraction despite the lack of preexisting periodontal disease on the distal side of the L2M.

Some studies aimed to prevent the formation of future periodontal defects around the L2M by grafting the extraction socket of the removed impacted L3M. In a study by Sammartino and his associates, they used autologous platelet-rich plasma to promote healing, which reduced the incidence of possible periodontal complications on the L2M [11]. In another investigation, the regeneration technique showed that the use of bovine porous bone mineral with or without a collagen membrane can be a viable and stable treatment to alleviate the periodontal defects that are often associated with impacted L3M extractions [12]. The best clinical results were noted in the bovine porous bone mineral plus collagen membrane group. A literature review concluded that flap design does not influence probing depth or attachment level on the distal aspect of the L2M following surgery of the L3M. Curettage of the radicular surface of L2M together with oral hygiene control reduces these clinical parameters. Bone regeneration techniques with bone graft are recommended in cases of prior periodontal defect distal to L2M [13].

Reports provide evidence of increased attachment levels when using GTR technique for the treatment of periodontal defects that occur on the distal aspect of L2M which are next to an extracted mesioangular impacted L3M with oral communication. A study was conducted by Oxford et al. to test if the use of GTR can enhance probing attachment levels following extraction of mesioangular impacted third molars. 12 patients with bilateral soft tissue impacted L3M entered this split mouth study. After extractions, the previously exposed distal root surface of L2M was debrided. The defects on the randomly selected experimental sites were covered with expanded polytetrafluoroethylene membrane and the tissue was replaced to cover the membrane. Membranes were removed after 6 weeks. Control sites were treated identically except that no membrane was placed. The use of barrier material did not provide statistically significant differences in probing attachment levels when comparing experimental versus control sites. Nevertheless, probing attachment levels gain was consistently greater at 3 and 6 months when GTR techniques were used in sites with deep impactions [6]. All the above procedures dealt with the advanced bone loss on the distal side of the L2M at the time of L3M extraction.

A randomized clinical trial was carried out to evaluate the healing of periodontal intrabony defects at the distal aspect of L2M using a resorbable polylactic acid barrier and a nonresorbable polytetrafluoroethylene barrier and to compare the therapeutic effect of the bioresorbable barrier versus the nonresorbable barrier. 19 patients with intrabony defects distal to L2M of > or =4 mm (on radiographs) were included in the study. The defects remained 5 years after surgical removal of impacted L3M. Following flap elevation and defect debridement, the defects were randomly covered with either a resorbable or a nonresorbable barrier. Treatment was evaluated clinically after 1 year by measurements of probing depth, probing attachment level, and probing bone level and radiographically by measurements of bone levels on radiographs taken immediately before and 1 year after surgery.

Both treatments resulted in significant pocket reduction, attachment gain, and radiographic bone fill. It was concluded that the GTR treatment of deep intrabony defects distal to L2M using resorbable barriers resulted in significant improvement at least equivalent to the results obtained using nonresorbable barriers [14]. The efficacy of platelets-rich plasma combined with bovine hydroxyapatite bone graft and resorbable membrane was investigated both clinically and radiographically for the treatment of intrabony pockets on the distal aspect of L2M following the surgical extraction of fully impacted L3M. The study concluded that the combined use of platelet-rich plasma with bovine hydroxyapatite bone graft materials for the treatment of intrabony defects might be an appropriate approach when the main goal is providing earlier bone regeneration [15]. Another combination that included platelet-rich plasma and a resorbable membrane of procaine origin (Bio-Gide; Geistlich Biomaterials, Wolhusen, Switzerland) was investigated in a randomized controlled trial in 2009. From a clinical point of view, it was not evident that the above-mentioned combination was superior to using platelet-rich plasma alone. However, histological analysis showed earlier onset of bone maturation [16].

In our present case, Bio-Oss was selected for its safety, excellent compatibility, and high porosity which can promote cell adhesion and differentiation. To enhance this effect, autogenous chips of bone were mixed with the Bio-Oss and the mix was covered with Bio-Gide barrier; to our knowledge this is the first case report of this kind using this mixture which resulted in a quicker healing time and excellent bone fill as can be seen in the postoperative X-ray.
